# Forecasting acute childhood malnutrition in Kenya using machine learning and diverse sets of indicators

**DOI:** 10.1371/journal.pone.0322959

**Published:** 2025-05-14

**Authors:** Girmaw Abebe Tadesse, Laura Ferguson, Caleb Robinson, Shiphrah Kuria, Herbert Wanyonyi, Samuel Murage, Samuel Mburu, Rahul Dodhia, Juan M. Lavista Ferres, Bistra Dilkina

**Affiliations:** 1 Microsoft AI for Good Research Lab, Nairobi, Kenya; 2 University of Southern California, Institute on Inequalities in Global Health, Los Angeles, California, United States of America; 3 Amref International University, Nairobi, Kenya; 4 Microsoft AI for Good Research Lab, Redmond, Washington, United States of America; 5 Amref Health Africa, Nairobi, Kenya; 6 Division of Nutrition and Dietetics, Ministry of Health, Nairobi, Kenya; 7 University of Southern California, Center for AI in Society, Los Angeles, California, United States of America; Addis Ababa University, ETHIOPIA

## Abstract

**Objectives:**

Malnutrition is a leading cause of morbidity and mortality for children under-5 globally. Low- and middle-income countries, such as Kenya, bear the greatest burden of malnutrition. The Kenyan government has been collecting clinical indicators, including on malnutrition, using District Health Information Software-2 (DHIS2) for over a decade. We aim to address the existing gap in decision-makers’ ability to develop and utilize malnutrition forecasting capabilities for timely interventions. Specifically, our objectives include: develop a spatio-temporal machine learning model to forecast acute malnutrition among children in Kenya using DHIS2 data, enhance forecasting capability by integrating external complementary indicators, such as publicly available satellite imagery-driven signals, and forecast acute malnutrition at various stages and time horizons, including moderate, severe, and aggregated cases.

**Methods:**

We propose a framework to forecast malnutrition risk for each sub-county in Kenya based on clinical indicators and remote sensory data. To achieve this, we first aggregate clinical indicators and remotely sensed satellite data, specifically gross primary productivity measurements, to the sub-county level. We then label the rate of children diagnosed with acute malnutrition at the sub-county level using the standard Integrated Food Security Phase Classification for Acute Malnutrition. We then apply and compare several methods for forecasting malnutrition risk in Kenya using data collected from January 2019 to February 2024. As a baseline, we used a Window Average model, which captures the current practice at the Kenyan Ministry of Health. We also trained machine learning models, such as Logistic Regression and Gradient Boosting, to forecast acute malnutrition risk based on observed indicators from prior months. Different metrics, mainly Area Under Receiver Operating Characteristic Curve (AUC), were used to evaluate the forecasting performance by comparing their forecast values to known values on a hold-out test set.

**Results:**

We found that machine learning based models consistently outperform the Window Average baselines on forecasting sub-county malnutrition rates in Kenya. For example, the Gradient Boosting model achieves a mean AUC of 0.86 when forecasting with a 6-month time horizon, compared to an AUC of 0.73 achieved by the Window Average model. The Window Average method particularly fails to correctly forecast malnutrition in parts of West and Central Kenya where the acute malnutrition rate is variable over time and typically less than 15%. We further found that machine learning models with satellite-based features alone also outperform Window Averaging baselines, while not needing clinical data at inference time. Finally, we found that recently observed outcomes and the remotely sensed data are key indicators. Our results demonstrate the ability of machine learning models to accurately forecast malnutrition in Kenya at a sub-county level from a variety of indicators.

**Conclusions:**

To the best of the authors’ knowledge, this work is the first to use clinical indicators collected via DHIS2 to forecast acute malnutrition in childhood at the sub-county level in Kenya. This work represents a foundational step in developing a broader childhood malnutrition forecasting framework, capable of monitoring malnutrition trends and identifying impending malnutrition peaks across more than 80 low- and middle-income countries collecting similar DHIS2 datasets.

## Introduction

In 2022, an estimated 45 million children under-5 worldwide were affected by acute malnutrition (wasting), of whom 13.6 million were suffering from severe wasting. This constitutes 6.8% and 2.1% of the global number of children under-5, respectively [1]. Malnutrition compromises a child’s immunity and is a significant cause of morbidity and mortality thereby impairing health outcomes across the child’s life course [1]. While food insecurity is a significant factor, malnutrition has been accelerated by population growth, climate change, economic crises, and conflicts [2–6].

The greatest burden of malnutrition is in low- and middle-income countries (LMICs), such as Kenya [7–9]. The 2022 Kenya Demographic Health Survey (DHS) found a 5% prevalence of acute malnutrition among children, a level considered a public health concern [10]. Policymakers at the Kenyan Ministry of Health (MoH) currently use historical trends in malnutrition to inform their efforts to address it, but there is no automation or statistical modeling that guides them.

Countries are increasingly using data- or evidence-based approaches to help understand malnutrition, including predictive machine learning (ML) models that allow for targeted interventions designed to mitigate malnutrition [11–15]. However, most of these models are built on demographic and socio-economic indicators collected through surveys that are limited in area coverage and collection frequency (e.g., DHS surveys are collected approximately every five years), limiting the ability of these approaches to forecast short-term changes in acute malnutrition [13, 16].

With increasingly complex and high-dimensional data becoming available in the field of nutrition, there is a need to use sophisticated methods such as ML for analysis [17]. Côté and Lamarche recently highlighted the potential of artificial intelligence (AI) in nutrition research, particularly in predicting health outcomes [11]. Naumova provided an overview of the challenges in understanding child malnutrition – without using specific predictive models, and highlighted the need to use diverse sets of indicators (including, for example, climate data) to make forecasting actionable [14].

Backer and Billing used a Random Forest algorithm to forecast the prevalence of acute child malnutrition in 36 sub-Saharan African countries by leveraging various sets of indicators, such as DHS, the Multiple Indicator Cluster Survey (MICS) and the Standardized Measurement of Relief and Transition (SMART) surveys. They showed that their approach could forecast acute malnutrition across different time horizons [15]. Similarly, Browne *et al*. demonstrated the potential of publicly available geo-referenced agricultural and geographic data to estimate malnutrition prevalence across multiple LMICs [18]. Bhavnani *et al*. recently studied comprehensive household surveys collected from selected Kenyan counties to develop and test a computational model examining the influence of household behavior variability on malnutrition vulnerability. The authors used the 5-point Integrated Food Security Phase Classification Acute Malnutrition (IPC-AMN) scale [19]. Other examples of indicators used to study malnutrition in Kenya include DHS and MICS [15], and even facial photography [20]. In Uganda, District Health Information Software-2 (DHIS2) [21] data have been used in a predictive model to establish associations between relevant climate-related data and the incidence of climate-sensitive diseases at specific study sites [22]. However, to the best of the authors’ knowledge, DHIS2 data have not been used to forecast acute malnutrition in children, nor has it been used in Kenya. This highlights an existing gap in decision-makers’ ability to develop and utilize malnutrition forecasting capabilities for timely and effective interventions.

The specific objectives of this study were to:

1. Develop a spatio-temporal machine learning model to forecast acute malnutrition among children in Kenya using DHIS2 data.
2. Enhance forecasting capability by integrating external complementary indicators, such as publicly available satellite imagery-driven signals.
3. Forecast acute malnutrition at various stages and time horizons, including moderate, severe, and aggregated cases.

## Material and methods

### Data

**Fig 1:**
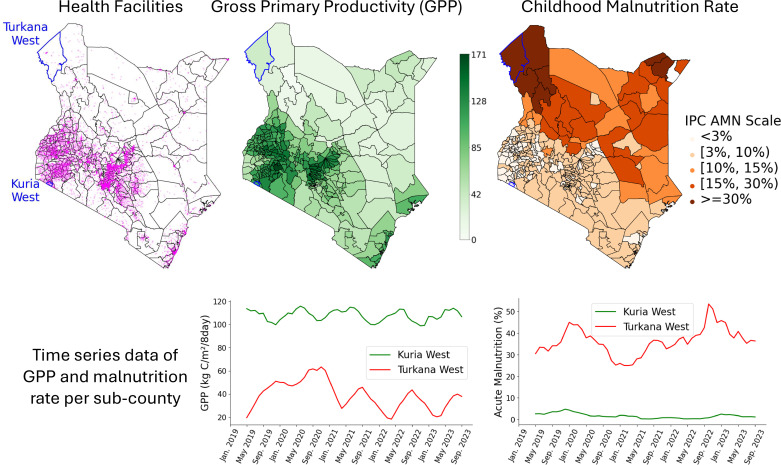
Health facilities where DHIS2 data were collected [top left]. The facilities are more concentrated in the western and central parts of Kenya, partly due to the denser population settlements in these regions. We also used remotely sensed data, i.e., averaged monthly Gross Primary Productivity (GPP) over the study period [top center]. Acute malnutrition is the target outcome, and its prevalence across Kenya, specifically the average monthly prevalence, is shown using the IPC AMN scale [top right]. At the bottom are sub-county examples of remotely sensed GPP values and malnutrition outcomes (in %) from the DHIS2 data for *Turkana West* and *Kuria West* sub-counties [bottom], which are known to have higher and lower malnutrition risks, respectively.

We constructed a framework that forecasts the risk of childhood acute malnutrition in each sub-county in Kenya using routinely collected health data obtained from DHIS2 as well as remotely sensed data obtained from satellites (see Fig 1). DHIS2 is an open-source, web-based platform for managing health information with the goal of improving healthcare delivery and decision-making [21]. The DHIS2 indicators were collected for the specific population group of children under the age of 5 years, and only the number of children is reported for each clinical indicator, i.e., there are no other metadata, such as sex, that could be used for stratification and subgroup analysis. The data were collected monthly across 17,324 geo-referenced health facilities in Kenya. We used monthly DHIS2 data, particularly malnutrition related clinical indicators, collected from January 2019 to February 2024 across 320 sub-counties in Kenya. More details on the diversity of data across counties and study period is shown in S1 Fig. Analysis of observed malnutrition outcomes.

#### Clinical indicators from DHIS2.

We extracted clinical indicators from DHIS2 including information on: infant feeding practices, diarrhea treatment, exclusive breastfeeding, pregnant women with low hemoglobin levels, low birth weight, underweight children, severely underweight children, length of stay in health facilities, recipients of different nutritional supplements and in-household food security program. The full list of the clinical indicators is shown in S1 Table. Full list of clinical indicators. As input to our forecasting models, we convert clinical indicators into monthly proportions using the total number of children visiting health facilities in each subcounty.

#### Target outcome.

For forecasting, we set the malnutrition risk as the target outcome, which is derived from the monthly rate of children who visited health facilities in the sub-county and who met the definition of acute (moderate to severe) malnutrition, as determined by the WHO’s child growth standards [23]. Our forecasting framework also offers the flexibility to analyze moderate acute malnutrition (MAM) and severe acute malnutrition (SAM) as separate target outcomes. This capability is valuable for understanding the specific risks associated with each stage of malnutrition and for advising targeted interventions accordingly.

#### Mapping the target outcome to the 5-point IPC acute malnutrition (AMN) scale.

We adopted the IPC AMN scale reported in [19] to map the monthly acute malnutrition rate to one of the five categories, i.e., [0,3%), [3,10%), [10,15%), [15,30%), and ≥30%. This provides a relative scale to compare malnutrition risks across sub-counties. This approach allows us to formulate our task as a 5-class forecasting problem. The stratification of the rates of acute malnutrition across all the sub-counties in the study period shows that ≈81% of the monthly observations experienced <10% acute malnutrition rate (see S2 Fig. Stratification across the IPC AMN scale). On the other hand, only ≈5% of these observations experienced ≥30% acute malnutrition rate. For the separate analysis of severe acute malnutrition, which is observed rarely compared to moderate acute malnutrition, we employed a modified IPC SAM scale as [0,0.5%), [0.5%,1%), [1,3%), [3,5%) and ≥5%. But we adopt the IPC AMN scale for the separate analysis of MAM.

#### Remotely sensed and publicly available data.

Given that food insecurity is a key driver of acute malnutrition [6], we incorporate remotely sensed data reflecting crop activity into our framework. The National Aeronautics and Space Administration’s (NASA’s) Moderate Resolution Imaging Spectro-radiometer (MODIS) is a satellite-based sensor used for earth and climate measurements. MODIS generates data on an 8-day cycle that can be used for agricultural monitoring. Derived variables from MODIS include Gross Primary Productivity (GPP) which measures the rate at which plants convert solar energy into chemical energy and provides a coarse indicator of crop health and productivity. We aggregated monthly MODIS GPP readings for each sub-county (averaged) in Kenya for the study’s period (see Fig 1). Lower average GPP values, e.g., in Northern and Eastern parts of Kenya, could be an early indication of food scarcity.

### Analysis and model development

#### Train-test split.

The data used in this study were collected from January 2019 to February 2024 resulting in 20,160 sub-county-based monthly samples for our analysis. Data collected between January 2019 and September 2023 formed the main study period and were used to train and test our models. Data collected between October 2023 and February 2024 were acquired later and used as an external validation set. Specifically, we split the available data in the main study period into *non-overlapping* train and test sets using ≈80% - 20% split. Data from each sub-county collected between January 2019 and September 2022 were used as the training set, while data collected between October 2022 and September 2023 were used as the hold-out test set.

#### Models.

**Fig 2:**
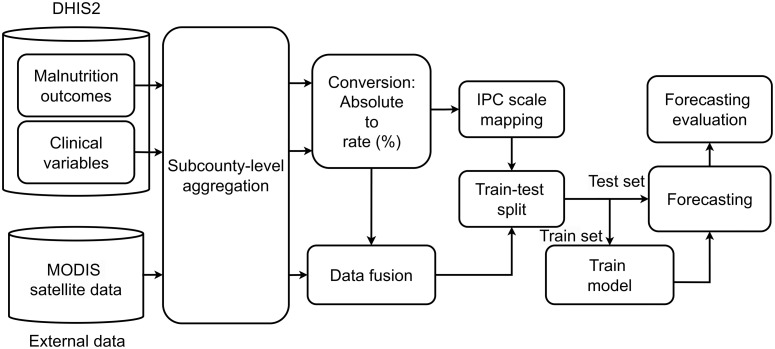
Our framework for forecasting acute malnutrition rates at the sub-county level in Kenya. Different sets of indicators are used to train the machine learning models, where the performance of the models is evaluated on a hold-out test set.

We tested three models in our forecasting framework (see Fig 2): Window Averaging (WA), Logistic Regression (LR), and Gradient Boosting (GB). The WA model forecasts future acute malnutrition risk by averaging previously observed acute malnutrition values and serves as our baseline model. LR and GB are ML models that, unlike WA, utilize the statistical relationships between a variety of indicators and the target outcomes. LR, a commonly used statistical model for classification tasks, is simple and easily interpretable but may underperform when non-linear relationships exist between the target and features. GB, an ensemble technique, sequentially builds decision trees to correct errors of previous ones and has demonstrated high performance on similar tasks [24]. Prior to model training, we adopted standardization of different sets of indicators, i.e., scaling continuous features by removing the mean and scaling to unit variance, whereas the categorical features were transformed using one-hot encoding.

#### Diverse sets of indicators as input to our models.

Given DHIS2 and GPP data, we extracted multiple indicators (and their combinations) as features to our models. The set of indicators include: PO (previous malnutrition outcomes), CF (clinical indicators from DHIS2 data), GPP (remotely sensed MODIS data on gross primary productivity), and S (sub-county indicator). We set the look-back horizon to three months. To reduce the number of model inputs and minimize potential overfitting, we use the mean value of each feature in the CF set across the past three months instead of using individual values. One-hot encoding was applied for the sub-county indicator feature (S).

#### Forecasting horizon.

We explored forecasting horizons that might be useful to policymakers seeking to intervene before an increase in malnutrition rates manifests. To this end, we used three forecast horizons: 1-month, 3-months and 6-months.

#### Evaluation metrics.

To evaluate the forecasting capabilities of our models, we used *Area Under Receiver Operating Characteristic Curve (AUC)* as the main metric due to its independence on a specific decision threshold. We also used *Accuracy* and *F*_*1*_*-score* (computed from *Precision* and *Recall*) to compare our approach with existing forecasting frameworks. A one-vs-all (OVA) strategy was applied to compute aggregated metrics across all five classes in the IPC AMN scale. The OVA strategy treats each class in a multiclass problem in a binary manner, where performance is evaluated for that class against all the remaining classes. The final performance metric is then computed as the average of the per-class values.

#### Uncertainty quantification.

We bootstrapped the test set 100 times to evaluate the uncertainty of our forecasting models. Performance metrics are presented as the mean of the results from the bootstrapped test sets, along with their standard deviations (STD) and 95% Confidence Intervals (CI). We also conducted paired t-tests to assess the statistical significance of the forecast improvements.

#### Software implementation.

We used a Python (version 3.9.19) programming language to implement our framework. We also used existing libraries of ML models, preprocessing and visualization tools from scikit-learn library (version 1.5.1) [25]. We also used Pandas library (version 2.2.2) [26] for data analysis, GeoPandas library [27] (version 0.14.2) and Matplotlib library [28] (version 3.8.4) for visualization.

### Ethics statement

The study utilized secondary data; there was no primary data collection from any human subjects. The DHIS2 data were anonymized and a waiver for informed consent was sought from the Amref Ethics and Scientific Research Committee (ESRC); the protocol was approved (certificate number ESRC P1 550/2023). Permission was sought from the National Commission for Science, Technology and Innovation (NACOSTI) and a license issued: NACOSTI/P/24/33659. All the investigators undertook relevant courses on protection of human research participants and all those who accessed the data signed a data confidentiality agreement. Only de-identified data were utilized.

## Results

### Forecasting acute malnutrition using DHIS2 data

Table 1 provides the performance of our forecasting framework tested across different sets of indicators, models and forecast horizons. Our models achieved a mean AUC of 0.89, 0.87 and 0.86, respectively, for 1-month, 3-months and 6-months horizons. The ML models, LR and GB, significantly outperformed the WA model at p-value threshold of 0.05 in a one-sided t-test (see S2 Table. Paired t-test for models’ comparison for t-test results across models using PO). Additionally, PO indicators demonstrated higher forecasting performance compared to CF indicators across the horizons.

**Table 1:** Indicators from DHIS2 are found to be able to forecast acute malnutrition. AUC results are presented as mean±std, [95% CI] derived from forecasting acute malnutrition across different forecast horizons using various sets of indicators and ML models. Indicators include PO (previous outcome), CF (clinical features), and S (sub-county indicator), with the “+” sign denoting the concatenation of indicators. The models assessed are WA (Window Average), LR (Logistic Regression), and GB (Gradient Boosting).

		Forecasting Horizon		
Indicator	Model	1-month	3-months	6-months
PO	WA	0.76 ± 0.01, [0.75, 0.77]	0.75 ± 0.01, [0.74, 0.76]	0.73 ± 0.01, [0.72, 0.74]
	LR	0.87 ± 0.00, [0.86, 0.88]	0.86 ± 0.01, [0.84, 0.86]	0.84 ± 0.01, [0.83, 0.85]
	GB	0.89 ± 0.00, [0.88, 0.90]	0.87 ± 0.01, [0.86, 0.88]	0.86 ± 0.01, [0.84, 0.87]
CF	LR	0.68 ± 0.01, [0.67, 0.69]	0.70 ± 0.01, [0.69, 0.71]	0.73 ± 0.01, [0.71, 0.74]
	GB	0.81 ± 0.01, [0.80, 0.82]	0.81 ± 0.01, [0.80, 0.82]	0.80 ± 0.01, [0.78, 0.81]
PO+CF	LR	0.86 ± 0.00, [0.86, 0.87]	0.86 ± 0.01, [0.85, 0.87]	0.84 ± 0.01, [0.83, 0.85]
	GB	0.88 ± 0.00, [0.88, 0.89]	0.87 ± 0.01, [0.86, 0.88]	0.85 ± 0.01, [0.84, 0.86]
PO+CF+S	LR	0.86 ± 0.01, [0.85, 0.87]	0.85 ± 0.01, [0.84, 0.86]	0.84 ± 0.01, [0.83, 0.86]
	GB	0.89 ± 0.00, [0.88, 0.89]	0.87 ± 0.01, [0.86, 0.88]	0.86 ± 0.01, [0.84, 0.87]

We analyzed the performance of the models across each level of the IPC AMN scale (see Table 2). All models achieved a high AUC (>0.9) across different horizons when forecasting extreme (≥30%) acute malnutrition risk. The WA model had the lowest performance, particularly in the [10% - 15%) range of acute malnutrition rate, where the ML models achieved ≈20% AUC improvement. Overall, the inferior performance of the WA model for the lower ranges (i.e., <15%) of the IPC AMN scale highlights its limitations for the sub-counties in Central, Western, and Southern Kenya (see Fig 1). The confusion matrix (shown in S3 Fig. Confusion matrix) demonstrates a clear distinction of the levels in the IPC AMN scale though adjacent levels tend to experience some misclassification, e.g., [3%,10%) and [10%,15%) particularly when PO indicators were used. This is partly due to the smaller number of training examples in [10%,15%) range (see S2 Fig. Stratification across the IPC AMN scale). The combination of multiple indicators reduces forecasting errors for [3%,10%) and [10%,15%) ranges (S3 Fig. Confusion matrix). Additionally, we validated the robustness of our framework against recent observations from October 2023 to February 2024. The results demonstrated the adequacy of our framework in forecasting acute malnutrition even a year after the model was trained (S4 Table. Model robustness).

**Table 2:** The ML models, Logistic Regression (LR) and Gradient Boosting (GB) models, outperformed Window Average (WA) model across the IPC AMN categories. All models achieved an AUC >0.9 for forecasting extreme malnutrition risk (≥30%). The WA model struggled in the lower ranges of the IPC AMN scale, particularly in the [10%, 15%) range, whereas the GB model performed consistently well across the ranges.

			IPC AMN 5-point scale				
Horizon	Model	Indicator	[0% , 3%)	[3% , 10%)	[10% , 15%)	[15% , 30%)	≥ 30%
1-month	WA	PO	0.77±0.01	0.74±0.01	0.64±0.01	0.75±0.01	0.91±0.01
	GB	PO	0.90±0.01	0.84±0.01	0.83±0.01	0.89±0.01	0.98±0.01
		CF	0.82±0.01	0.75±0.01	0.71±0.01	0.81±0.01	0.95±0.01
		PO+CF	0.90±0.01	0.83±0.01	0.82±0.01	0.88±0.01	0.98±0.01
		PO+CF+S	0.90±0.01	0.84±0.01	0.82±0.01	0.88±0.01	0.98±0.01
3-months	WA	PO	0.76±0.01	0.73±0.01	0.61±0.01	0.74±0.01	0.91±0.01
	GB	PO	0.89±0.01	0.83±0.01	0.80±0.02	0.88±0.01	0.98±0.01
		CF	0.83±0.01	0.75±0.01	0.71±0.02	0.83±0.01	0.94±0.01
		PO+CF	0.89±0.01	0.83±0.01	0.79±0.01	0.86±0.01	0.97±0.01
		PO+CF+S	0.90±0.01	0.84±0.01	0.80±0.01	0.85±0.01	0.98±0.01
6-months	WA	PO	0.76±0.01	0.72±0.01	0.59±0.01	0.70±0.02	0.90±0.02
	GB	PO	0.87±0.00	0.81±0.01	0.78±0.01	0.85±0.01	0.98±0.01
		CF	0.81±0.01	0.74±0.01	0.67±0.02	0.81±0.02	0.94±0.01
		PO+CF	0.88±0.01	0.80±0.01	0.76±0.02	0.84±0.02	0.98±0.00
		PO+CF+S	0.88±0.01	0.81±0.01	0.78±0.02	0.84±0.02	0.98±0.01

### Complementing DHIS2 data with remotely sensed GPP data

**Fig 3:**
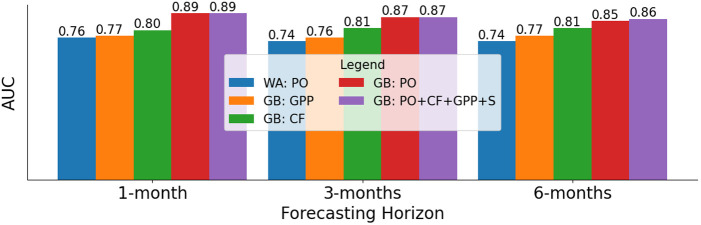
Gradient boosting (GB) model trained with remotely sensed GPP data (GB: GPP) slightly outperformed the Window Average baseline (WA: PO), particularly in 3- and 6-months forecast horizons. GPP also achieved competitive performance (in AUC) with the clinical features (CF) in DHIS2. PO: previous outcome, S: Subcounty indicator.

**Fig 4:**
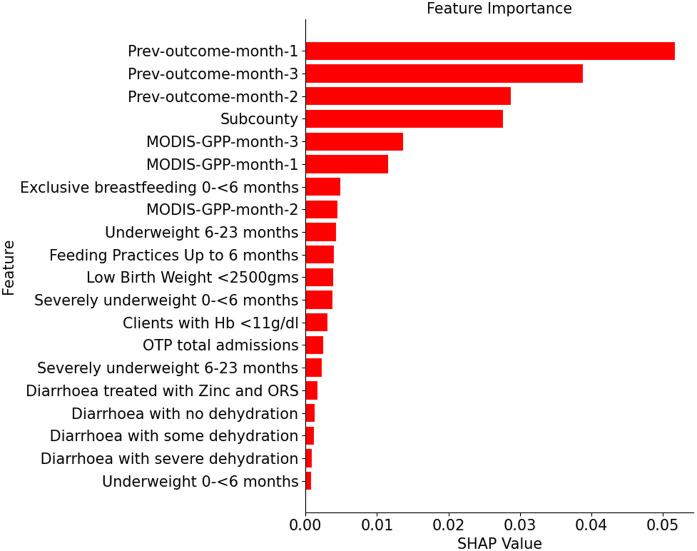
Indicators comprising of previous outcomes (PO) are ranked top on feature importance scores based on SHAP values computed for a Gradient Boosting (GB) model for 3-months forecast horizon. Notably, features derived from GPP are scored competitively with the clinical features (CF) from DHIS2. The SHAP value for the categorical Subcounty feature is computed as the aggregated sum of the values for the corresponding one-hot vector elements.

The GPP indicators extracted from remotely sensed data performed competitively with CF in DHIS2 data (see Fig 3). Notably, GPP-trained GB model alone outperformed the baseline WA, particularly in forecasting with 6-months horizon, i.e., 0.74 vs. 0.77 AUC (see Fig 3). Furthermore, GPP alone slightly outperformed the WA baseline, particularly in forecasting ≥10% acute malnutrition rates (see S3 Table. GPP’s impact). In our analysis of features’ importance for the best performing ML model (GB) (see Fig 4), PO indicators (e.g., *Prev-outcome-month-1, -2, -3*) ranked top, followed by the sub-county indicator. GPP-derived features (e.g., *MODIS-GPP-month-3*) ranked higher than clinical features from DHIS2. This further validates the complementary potential of the publicly available and remotely sensed GPP data, particularly when there are no PO and/or CF indicators. Once trained, a GPP-only ML model can be used to forecast without further access to DHIS2 information.

### Separate forecasting of MAM and SAM

We also used our framework to forecast the risks of moderate (MAM) and severe (SAM) acute malnutrition separately. The forecasting results for MAM exhibited a pattern very similar to the results for acute malnutrition presented earlier. Forecasting SAM, on the other hand, is relatively challenging due to its rare occurrences in the training data. Nevertheless, both LR and GB achieved AUC values exceeding >0.8 across all the forecast horizons (see S5 Table. Aggregated forecast of severe acute malnutrition). The GB model outperformed the WA baseline across the five-point modified IPC SAM scale (see S6 Table. Forecast of severe acute malnutrition using IPC SAM scale). Both WA and GB models struggled to forecast when the SAM risk is in the low [0.5%, 1%) range.

## Discussion

DHIS2 data have the potential to inform governments’ and civil society organizations’ work on nutrition by forecasting the risk of child malnutrition across the sub-counties in Kenya using ML models. This is particularly critical both in Kenya and across all Sub-Saharan Africa, where the recovery rate from severe acute malnutrition among children remains low [29]. Although the WA model (which is close to the current practices employed on the ground) offers simplicity, the ML models, such as LR and GB, consistently delivered higher forecast performance. Models performed well even during forecasting for longer time horizons, but with slightly lower value, e.g., mean AUC of 0.86 for 6-months compared to 0.89 for 1-month. This is partly due to the potential temporal drift or the limitation of the smaller time period that is limited for identifying seasonal variations and long-term trends. Thus, the performance might degrade further if the models were used to forecast too far in the future.

While there are similar works in forecasting acute malnutrition at individual-, village- or country-levels, often using DHS data, our work provides a competitive forecasting performance but at sub-county level in Kenya. For example, Backer and Billing reported acute malnutrition forecasting with test set accuracy of 0.59 to 0.63 between 1- to 12-months horizon using multiple household surveys (e.g., DHS, MICS and SMART) [15], whereas our framework achieved a test accuracy of 0.69 to 0.72 between 1- to 6-months horizon using the GB model trained on DHIS2 and remotely sensed GPP data. Bhavnani et. al. reported *F*_1_ = 0.51 for ward-level forecasting of acute malnutrition with 12-months horizon using the IPC AMN scale [19]. Although direct comparisons are challenging due to differences in the sets of used indicators and the administrative levels for which forecasts are made, our framework achieved an *F*_1_ score of 0.58±0.01 for a sub-county-based forecast with a horizon of 6-months.

The only remotely sensed indicator (i.e., GPP) achieved competitive forecasting performance that remained consistent across different forecast horizons. For example, GPP alone achieved an AUC of 0.77, 0.76, and 0.77 for 1-, 3-, and 6-month forecasting horizons, respectively. This is competitive with using DHIS2 clinical features, which achieved an AUC of 0.80, 0.81, and 0.81, respectively (see Fig 3). This suggests that such data could be used to extend forecasting capabilities in areas where the availability of the DHIS2 data is limited. Furthermore, GPP data provide a scalable, cost-effective alternative for decision-making, enhancing the reach and utility of our forecasting framework.

The previous outcome indicators were found to be the top-ranked predictors of future malnutrition risk as expected (Fig 4). GPP indicators ranked higher than clinical indicators extracted from DHIS2. Thus, GPP data could be utilized where DHIS2 data, particularly previous outcomes and clinical indicators were not available for forecasting or where concerns exist about the completeness or quality of the DHIS2 data.

Different latency periods exist between the indicators and observed acute malnutrition, so the combined use of various sets of indicators enhances the robustness of the framework across forecast horizons. Our framework provided competitive forecast performance across these horizons. Table 1 shows the AUC performance of 0.89, 0.87, and 0.86 for combined DHIS2 indicators in forecasting acute malnutrition across 1-, 3-, and 6-month horizons, respectively. Our approach can be easily calibrated for new forecast horizons, such as 12 months. The framework can be used in the future with minimal retraining efforts, as competitive performance was achieved with our model even a year after it was trained (S4 Table. Model robustness). Additionally, our framework is flexible enough to forecast severe and moderate acute malnutrition risks separately, in addition to aggregated acute malnutrition.

When decision-makers are given one to six months’ notice of where acute malnutrition is likely to occur, there is an opportunity for timely interventions that can make acute malnutrition temporary and reversible [15, 30]. Interventions can aim to improve dietary intake (nutrition-specific interventions) and prevent illness (nutrition-sensitive interventions), which increases nutrient needs while also impeding nutrient absorption [30]. Nutrition-specific interventions include supplementary feeding and support for pregnant women, infants, and young children. Nutrition-sensitive interventions encompass water, sanitation, and hygiene improvements, livelihoods support (e.g., cash transfers), and other similar measures that address the underlying causes of disease. The appropriate mix of interventions is context specific. Having additional time to intervene, before acute malnutrition emerges, may facilitate a multi-sectoral response, including governmental and humanitarian organizations, that includes both nutrition-specific and nutrition-sensitive interventions. Adopting the IPC AMN scale enables standardized quantification of acute malnutrition, thereby facilitating coordination among stakeholders. In resource-limited contexts, knowing how to efficiently target these interventions ensures the best use of available resources for maximum impact. In the Kenyan context, providing forecasts to the Nutrition Division within the Ministry of Health and the Nutrition Information Technical Working Group [31] can inform planning and resource allocation. The collaborative efforts between AI scientists, data analysts, social scientists, and nutrition experts from academia, civil society, government, and the private sector underscore the importance of multi-disciplinary approaches to solving complex health challenges such as acute malnutrition.

Work is ongoing with the Kenyan Ministry of Health to co-create a system that will allow them and their partners to receive these forecasts frequently. This eases interaction between the forecasting framework and decision-makers, thereby informing resource allocation for preventing and mitigating the impacts of acute malnutrition among children. It will be essential to assess, over time, how these forecasts have affected the national response to acute malnutrition. Furthermore, while our principal objective was to assess the suitability of DHIS2 for forecasting acute malnutrition among children under five years of age, future work could consider incorporating other sets of indicators beyond GPP data, such as rainfall, crop yields, or crop prices. Moreover, DHIS2 is designed to be adaptable to various health program areas, including malaria and HIV/AIDS, suggesting that a similar approach could be used to address other public health concerns.

## Limitations

Data is the critical component of trustworthy ML applications [32, 33]. Thus, the main limitation of this work derives from the DHIS2 data used to train our forecasting models. First, DHIS2 data were collected at the health facility level, potentially excluding children who were unable to visit health facilities for various reasons, including economic challenges and socio-cultural preferences for alternative care-seeking. Second, there is latency between the monthly reporting of DHIS2 data and the occurrence of acute malnutrition. Further study is required to analyze these time lags across the sets of indicators. Third, it is unclear whether zero values in the data are due to missing data (i.e., a failure to report) or the absence of acute malnutrition in the children who visited the facility. This issue requires further study, as well as ongoing efforts to ensure the quality of all health data in DHIS2. Fourth, there is a mismatch in administrative boundaries upon which DHIS2 data and GPP were collected. DHIS2 data were collected across 320 sub-counties, whereas the publicly available administrative boundary data [34], used for aggregating GPP measurements, consists of only 290 sub-counties. Reasons for the mismatch include the merging or creation of sub-counties in Kenya in recent years. Thus, we utilized 240 common sub-counties between the two administration boundary files for the experiments where GPP is included (Fig 3, Fig 4 and S3 Table. GPP’s impact). All the other experiments used the DHIS2 data from the 320 sub-counties. Though our experimental results suggest a low likelihood of model degradation in forecasting over longer time horizons (see Table 1, Table 2 and Fig 3) or when the time period of the training data is varied (see S4 Table. Model robustness), we acknowledge the potential limitation of training our models on historical data and validating them with more recent data, e.g., due to temporal dataset shift. While our current implement adopts a look-back horizon of three months, it could be further improved by utilizing a longer horizon to capture seasonal variations and long-term trends, especially when more historical data becomes available.

## Conclusions

This work serves as a proof-of-concept for employing machine learning and data-driven techniques to forecast acute malnutrition among children using DHIS2 and other publicly available data. This approach can encourage similar efforts in approximately 125 countries where DHIS2 is utilized, particularly in the 80 low- and middle-income countries where malnutrition remains a leading cause of child morbidity and mortality. The flexibility of our forecasting framework includes the capability to utilize different sets of indicators, such as DHIS2 and satellite-driven GPP signals, to provide analysis at the required administrative level. Furthermore, our framework is designed to forecast across different time horizons and can analyze severe and moderate acute malnutrition both collectively (as acute malnutrition) and separately. Providing lead time for addressing acute malnutrition among children can help redirect scarce resources to achieve maximum effect, ultimately saving lives.

## Supporting information

S1 Table Full list of clinical indicators. The observation rate per indicator is computed across months in the study period for each sub-county and then averaged across all the sub-counties.
(TIFF)

S2 Table Paired t-test for models’ comparison. Paired t-test results reflecting the statistical significance of forecast improvements by machine learning models. Compared to the Window Average (WA) baseline, the Logistic regression (LR) and Gradient Boosting (GM) models significantly outperformed the WA as shown in their t-test results (statistic, p-value) when the previous outcome (PO) set of indicators are used.
(TIFF)

S1 Fig Analysis of observed malnutrition outcomes. (a) Across all the 47 counties in Kenya. We computed the mean observation rate over all health facilities and months in the main study period for each county. Note that the counties with the highest and lowest observation rates are Makueni (0.71) and Nyeri (0.20), respectively. The mean observation rate across all the counties is 0.44. (b) The prevalence rate of acute malnutrition across counties computed across its subcounties. Turkana county has the highest rate (0.38), Siaya county the lowest (0.02), with an average prevalence across counties of 0.08. (c) Observation pattern of different types of outcomes over the collection period averaged over the health facilities. Note the similar patterns of the outcomes between the training set (January 2019 and September 2022) and the test set (October 2022 to September 2023). (d) The distribution of health facilities across counties. Nairobi county has the highest number of health facilities (1429), Lamu county the lowest (92), with an average across counties of 369.
(TIFF)

S2 FigStratification across the IPC AMN scale.The majority (≈81%) of observed acute malnutrition rates are below 10% in a given month.(TIFF)

S3 Fig Confusion matrix. Our models identified categories of the IPC AMN scale where aggregation of multiple indicators helped to improve performance for forecasting the [10%, 15%) range. Confusion matrices for forecasting acute malnutrition in Kenya with a 3-months forecast horizon: (a) using the Window Average model (WA) on the previous outcome (PO), (b) using Gradient Boosting (GB) on PO, (c) using GB on all the indicators in DHIS2 (PO+CF+S), and (d) using GB on all indicators in DHIS2 and GPP data (PO+CF+GPP+S). The “+" sign indicates the concatenation of different sets of indicators.
(TIFF)

S3 TableGPP’s impact.Gradient Boosting (GB) model, trained on the remotely sensed GPP data alone, achieved superior performance (in AUC) than the Window Average (WA) baseline, particularly for risk of acute malnutrition above 10% in 3-months horizon. Indicators include previous outcomes (PO), clinical features (CF), and sub-county indicator (S). The “+" sign indicates the concatenation of different sets of indicators. GPP performed competitively with CF from DHIS2.(TIFF)

S4 Table Model robustness. Our forecasting framework demonstrated robustness by consistently delivering accurate forecasting performance (in AUC) even a year after the model was trained. Validation of our forecasting models with recent DHIS2 data (October 2023 to February 2024). We employed two types of models based on the training periods: January 2019 to September 2022 and January 2019 to September 2023. Indicator sets include previous outcomes (PO), clinical features (CF), and sub-county indicators (S). The models evaluated were WA (Window Average), LR (Logistic Regression), and GB (Gradient Boosting). The “+" sign indicates the concatenation of different sets of indicators.
(TIFF)

S5 Table Aggregated forecast of severe acute malnutrition. Gradient Boosting (GB) model achieved higher performance (in AUC) than the Window Average (WA) and Logistic Regression (LR) to forecast severe acute malnutrition (SAM) across different horizons. The indicator sets include previous outcomes (PO), clinical features (CF), and sub-county indicators (S), with the “+” sign indicating the concatenation of different sets. Other models include WA: Window Average, LR: Logistic Regression.
(TIFF)

S6 Table Forecast of severe acute malnutrition using IPC SAM scale. Comparison of AUC results between WA (Window Average) and GB (Gradient Boosting) models using indicators from the previous outcomes (PO), where GB model is consistently the higher performing model than the WA model across the modified 5-point IPC SAM scale.
(TIFF)

## References

[1] World Health Organisation (WHO). UNICEF/WHO/The World Bank: Joint child malnutrition estimates (JME); 2023. Available from: https://www.who.int/teams/nutrition-and-food-safety/monitoring-nutritional-status-and-food-safety-and-events/joint-child-malnutrition-estimates (accessed: April 5, 2025).

[2] Bora S, Ceccacci I, Delgado C, Townsend R. Food security and conflict; 2011. Available from: https://openknowledge.worldbank.org/handle/10986/27245 (accessed: April 5, 2025).

[3] Hanjra MA, Qureshi ME. Global water crisis and future food security in an era of climate change. Food Policy. 2010;35(5):365–77. doi: 10.1016/j.foodpol.2010.05.006

[4] Misra AK. Climate change and challenges of water and food security. Int J Sustain Built Environ. 2014;3(1):153–65. doi: 10.1016/j.ijsbe.2014.04.006

[5] Homeida A. The complexities of conflict-induced severe malnutrition in Sudan. BMJ Glob Health. 2023;8(12):e014152. doi: 10.1136/bmjgh-2023-014152 38114238 PMC10749032

[6] Foini P, Tizzoni M, Martini G, Paolotti D, Omodei E. On the forecastability of food insecurity. Sci Rep. 2023;13(1):2793. doi: 10.1038/s41598-023-29700-y 36928341 PMC10038988

[7] Fanzo J. Healthy and sustainable diets and food systems: the key to achieving sustainable development Goal 2? Food Ethics. 2019;4(2):159–74. doi: 10.1007/s41055-019-00052-6

[8] (IFPRI) IFPRI. Global nutrition report 2015: Africa brief; 2015. https://ebrary.ifpri.org/digital/collection/p15738coll2/id/129780 (accessed: April 5, 2025).

[9] India State-Level Disease Burden Initiative Malnutrition Collaborators. The burden of child and maternal malnutrition and trends in its indicators in the states of India: the Global Burden of Disease Study 1990-2017. Lancet Child Adolesc Health. 2019;3(12):855–70. doi: 10.1016/S2352-4642(19)30273-1 31542357 PMC6839043

[10] Kenya National Bureau of Statistics TDPI Ministry of Health. Kenya Demographic and Health Survey 2022. 2023. Available from: https://www.knbs.or.ke/wp-content/uploads/2023/07/Kenya-DHS-2022-Main-Report-Volume-2.pdf (accessed: April 5, 2025).

[11] Côté M, Lamarche B. Artificial intelligence in nutrition research. Artif Intell Clin Practice. 2024:465–73. doi: 10.1016/b978-0-443-15688-5.00031-0

[12] Turjo EA, Rahman MH. Assessing risk factors for malnutrition among women in Bangladesh and forecasting malnutrition using machine learning approaches. BMC Nutr. 2024;10(1):22. doi: 10.1186/s40795-023-00808-8 38303093 PMC10832135

[13] Bitew FH, Sparks CS, Nyarko SH. Machine learning algorithms for predicting undernutrition among under-five children in Ethiopia. Public Health Nutr. 2022;25(2):269–80. doi: 10.1017/S1368980021004262 34620263 PMC8883776

[14] N Naumova E. Forecasting seasonal acute malnutrition: setting the framework. Food Nutr Bull. 2023;44(2_suppl):S83–93. doi: 10.1177/03795721231202238 37850923

[15] Backer D, Billing T. Forecasting the prevalence of child acute malnutrition using environmental and conflict conditions as leading indicators. World Dev. 2024;176:106484. doi: 10.1016/j.worlddev.2023.106484

[16] Ndagijimana S, Kabano IH, Masabo E, Ntaganda JM. Prediction of stunting among under-5 children in Rwanda using machine learning techniques. J Prev Med Public Health. 2023;56(1):41–9. doi: 10.3961/jpmph.22.388 36746421 PMC9925281

[17] Kirk D, Kok E, Tufano M, Tekinerdogan B, Feskens EJM, Camps G. Machine learning in nutrition research. Adv Nutr. 2022;13(6):2573–89. doi: 10.1093/advances/nmac103 36166846 PMC9776646

[18] Browne C, Matteson DS, McBride L, Hu L, Liu Y, Sun Y, et al. Multivariate random forest prediction of poverty and malnutrition prevalence. PLoS One. 2021;16(9):e0255519. doi: 10.1371/journal.pone.0255519 34495951 PMC8425567

[19] Bhavnani R, Schlager N, Donnay K, Reul M, Schenker L, Stauffer M, et al. Household behavior and vulnerability to acute malnutrition in Kenya. Humanit Soc Sci Commun. 2023;10(1):63. doi: 10.1057/s41599-023-01547-8 36811115 PMC9936478

[20] Watkins B, Odallo L, Yu J. Artificial intelligence for the practical assessment of nutritional status in emergencies. Expert Syst. 2024;41(7). doi: 10.1111/exsy.13550

[21] DHIS2. Digital Health Information Software. 2024. https://dhis2.org/ (accessed: April 5, 2025).

[22] Kaddu J, Gebru B, Kibaya P, Munabi I. Climate change and health in sub-Saharan Africa: The case of Uganda; 2020. Climate Investment Funds. https://www.climateinvestmentfunds.org/sites/cif_enc/files/knowledge-documents/climate_change_and_health_in_ssa_the_case_of_uganda_final.pdf (accessed: April 5, 2025).

[23] World Health Organisation (WHO). Child Growth Standards; 2009. Available from: https://www.who.int/publications/i/item/ (accessed: April 5, 2025).

[24] Westerveld JJL, van den Homberg MJC, Nobre GG, van den Berg DLJ, Teklesadik AD, Stuit SM. Forecasting transitions in the state of food security with machine learning using transferable features. Sci Total Environ. 2021;786:147366. doi: 10.1016/j.scitotenv.2021.147366 33971600

[25] Pedregosa F, Varoquaux G, Gramfort A, Michel V, Thirion B, Grisel O, et al. Scikit-learn: Machine learning in Python. J Mach Learn Res. 2011;12:2825–30. doi: 10.5555/1953048.2078195

[26] McKinney W. Data Structures for Statistical Computing in Python. In: van der Walt S, Millman J, editors. Proceedings of the 9th Python in Science Conference; 2010. pp. 51–56.

[27] Jordahl K, contributors G. GeoPandas: Python tools for geographic data; 2014–2023. https://geopandas.org/ (accessed: April 5, 2025).

[28] Hunter JD. Matplotlib: a 2D graphics environment. Comput Sci Eng. 2007;9(3):90–5. doi: 10.1109/mcse.2007.55

[29] Desyibelew HD, Bayih MT, Baraki AG, Dadi AF. The recovery rate from severe acute malnutrition among under-five years of children remains low in sub-Saharan Africa. A systematic review and meta-analysis of observational studies. PLoS One. 2020;15(3):e0229698. doi: 10.1371/journal.pone.0229698 32187182 PMC7080262

[30] de Pee S, Grais R, Fenn B, Brown R, Briend A, Frize J, et al. Prevention of acute malnutrition: distribution of special nutritious foods and cash, and addressing underlying causes—what to recommend when, where, for whom, and how. Food Nutr Bull. 2015;36(1 Suppl):S24-9. doi: 10.1177/15648265150361S104 25902611

[31] NISTWG. Nutrition Information System Technical Working Group; 2024. Avaialble from: https://www.nutritioncluster.net/sites/nutritioncluster.com/files/2020-06/Generic

[32] Oala L, Maskey M, Bat-Leah L, Parrish A, Gürel NM, Kuo TS, et al. DMLR: data-centric machine learning research-past, present and future. J Data-centric Mach Learn Res. 2024;doi: 10.48550/arXiv.2311.13028

[33] Liang W, Tadesse GA, Ho D, Fei-Fei L, Zaharia M, Zhang C, et al. Advances, challenges and opportunities in creating data for trustworthy AI. Nat Mach Intell. 2022;4(8):669–77. doi: 10.1038/s42256-022-00516-1

[34] United Nations Office for the Coordination of Humanitarian Affairs OCHA. Kenya - subnational administrative boundaries, 2023. https://data.humdata.org/dataset/cod-ab-ken (accessed: April 5, 2025).

