# Supplementary figures and images for: Forecasting acute childhood malnutrition in Kenya using machine learning and diverse sets of indicators
Source: PLoS One. 2025 May 14;20(5):e0322959. doi: 10.1371/journal.pone.0322959 (PMC12077733; doi:10.1371/journal.pone.0322959)

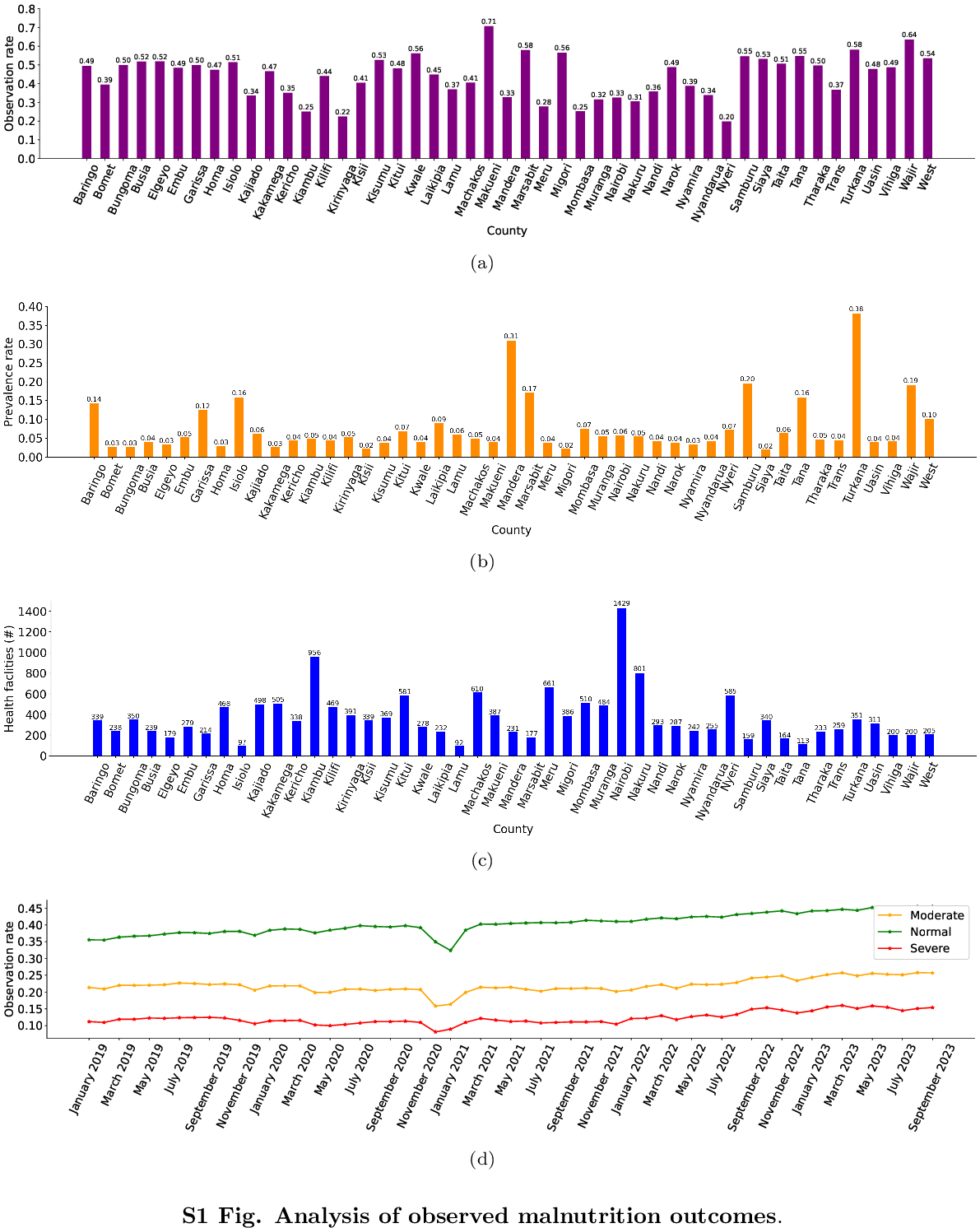

Supplement: S1 Fig — (a) Across all the 47 counties in Kenya. We computed the mean observation rate over all health facilities and months in the main study period for each county. Note that the counties with the highest and lowest observation rates are Makueni (0.71) and Nyeri (0.20), respectively. The mean observation rate across all the counties is 0.44. (b) The prevalence rate of acute malnutrition across counties computed across its subcounties. Turkana county has the highest rate (0.38), Siaya county the lowest (0.02), with an average prevalence across counties of 0.08. (c) Observation pattern of different types of outcomes over the collection period averaged over the health facilities. Note the similar patterns of the outcomes between the training set (January 2019 and September 2022) and the test set (October 2022 to September 2023). (d) The distribution of health facilities across counties. Nairobi county has the highest number of health facilities (1429), Lamu county the lowest (92), with an average across counties of 369. (TIFF) [file pone.0322959.s003.tif]

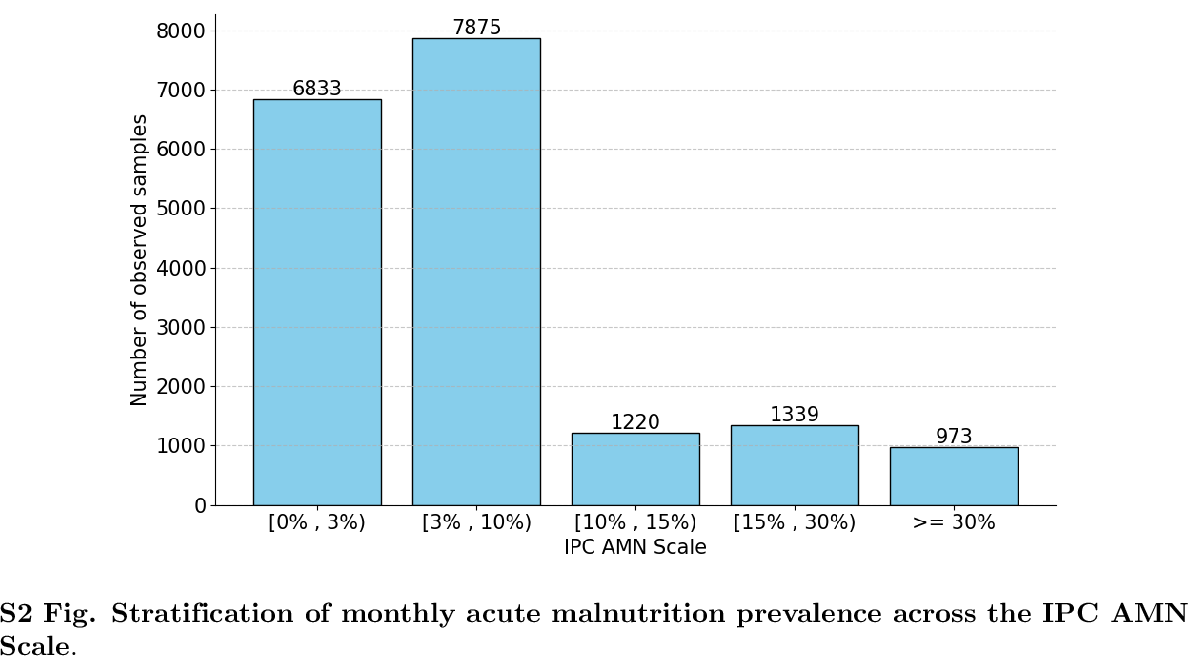

Supplement: S2 Fig — The majority (≈81%) of observed acute malnutrition rates are below 10% in a given month. (TIFF) [file pone.0322959.s004.tif]

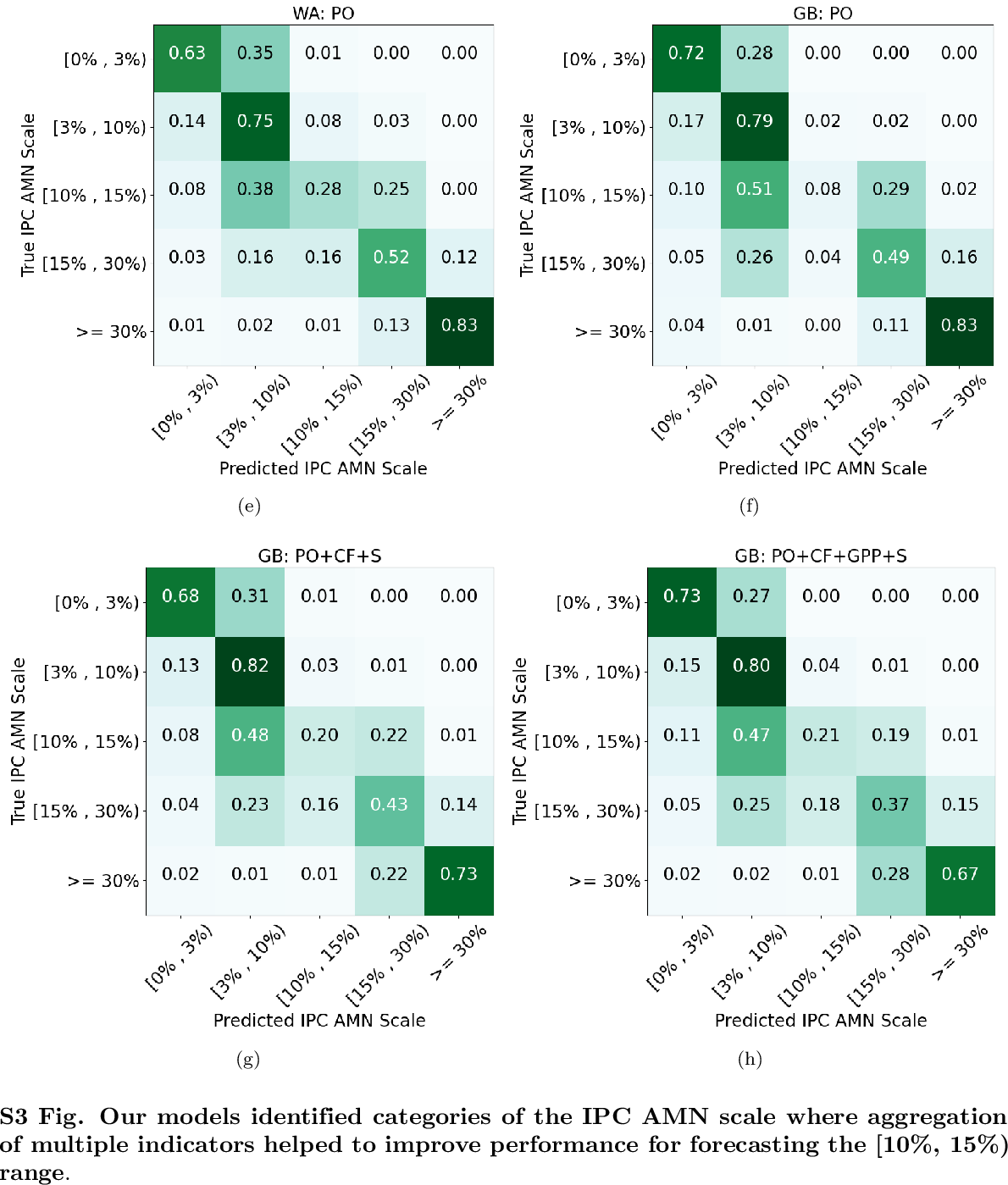

Supplement: S3 Fig — Our models identified categories of the IPC AMN scale where aggregation of multiple indicators helped to improve performance for forecasting the [10%, 15%) range. Confusion matrices for forecasting acute malnutrition in Kenya with a 3-months forecast horizon: (a) using the Window Average model (WA) on the previous outcome (PO), (b) using Gradient Boosting (GB) on PO, (c) using GB on all the indicators in DHIS2 (PO+CF+S), and (d) using GB on all indicators in DHIS2 and GPP data (PO+CF+GPP+S). The “+" sign indicates the concatenation of different sets of indicators. (TIFF) [file pone.0322959.s005.tif]
